# Side Effects and Perceptions of COVID-19 Vaccination in Saudi Arabia: A Cross-Sectional Study

**DOI:** 10.3389/fmed.2022.899517

**Published:** 2022-06-07

**Authors:** Mohammed Khaled Al-Hanawi, Mpho Keetile, Nasser Akeil Kadasah, Noor Alshareef, Ameerah M. N. Qattan, Omar Alsharqi

**Affiliations:** ^1^Department of Health Services and Hospital Administration, Faculty of Economics and Administration, King Abdulaziz University, Jeddah, Saudi Arabia; ^2^Health Economics Research Group, King Abdulaziz University, Jeddah, Saudi Arabia; ^3^Department of Population Studies, University of Botswana, Gaborone, Botswana; ^4^Department of Business Administration, Faculty of Economics and Administration, King Abdulaziz University, Jeddah, Saudi Arabia

**Keywords:** COVID-19, perceptions, Saudi Arabia, side effects, vaccination

## Abstract

**Background:**

Vaccination against any disease is critical in improving and maintaining public health. However, the overall effectiveness of a vaccine largely depends on the willingness of a population to receive it. The main aim of this study was to assess the side effects and perceptions about COVID-19 vaccines among adults following vaccination in Saudi Arabia.

**Methods:**

An online cross-sectional survey was conducted from July 13 to July 20, 2021, among adults aged 18 years and older who had taken one or both doses of COVID-19 vaccines in Saudi Arabia. The survey included questions on socio-demographics, health behavior, vaccine type, knowledge about sources of information about COVID-19 vaccines, and perceptions and beliefs following vaccination. Bivariate and multivariable regression analyses were the major data analytic tools employed in the study.

**Results:**

The most common vaccine side effects reported were tiredness/fatigue (52.6%), swelling (38%), fever (31.3%), headache (29.1%), and muscle pain (22.2%). In multivariable analyses, the odds of experiencing severe side effects were significantly higher among males [adjusted odds ratio (aOR) = 2.76, 95% confidence interval (CI) = 1.71–4.45, *p* < 0.01], those aged 40–49 years (aOR = 3.10, 95% CI = 1.10–8.72, *p* < 0.1), and Saudi nationals (aOR = 3.64, 95% CI = 1.58–8.38, *p* < 0.05) compared to their counterparts. The odds of believing that COVID-19 vaccines are safe in the long-term were significantly higher among men (aOR = 1.76, 95% CI = 1.16–2.65, *p* < 0.01) and among individuals who had received two doses (aOR = 1.62, 95% CI = 1.09–2.40, *p* < 0.05), and the odds of advising others to get vaccinated for COVID-19 were also significantly higher among respondents who had received two doses (aOR = 2.81, 95% CI = 1.60–4.93, *p* < 0.01) compared to their counterparts.

**Conclusion:**

This study identified the most common COVID-19 vaccine side effects in Saudi Arabia, therefore making them predictable. This information will help reduce vaccine hesitancy as booster doses become available.

## Introduction

The advent COVID-19 pandemic brought about enduring consequences to the health, economy and people's social lives across the globe (1). The pandemic has caused an enormous burden of illness worldwide, and several vaccines have been introduced to reduce morbidity and mortality. Moreover, the latest estimates indicate that over 20 million years of life have been lost to COVID-19 so far, and millions of new cases of COVID-19 are still being recorded every week despite the introduction of vaccines in many countries across the world (2).

Vaccination against any disease is very critical in improving and maintaining public health. Vaccines help to control the transmission of infectious diseases; however, the overall effectiveness of a vaccine largely depends on the willingness of the population to receive it (3–5). A global survey on the potential acceptance of COVID-19 vaccines showed varying acceptance rates among countries, ranging from almost 90% in China to <55% in Russia (6). In Australia (7), a study indicated that 80% of respondents generally held positive views toward COVID-19 vaccination while in Chile, a study on COVID-19 vaccine perception showed that about 91% of the sampled population were willing to be vaccinated (8). On the other hand, a study conducted in the Kingdom of Saudi Arabia (KSA) showed that only 48% of the Saudi population were willing to receive the COVID-19 vaccine (9).

Moreover, studies conducted to assess the acceptance, perceptions and attitudes of people toward COVID-19 vaccines have shown mixed results on the perceptions of people about COVID-19 vaccines and the factors influencing uptake of vaccines. Several factors have been observed to influence the uptake of vaccination, including perceptions about vaccine effectiveness and side effects, attitudes toward vaccination, perceived susceptibility to an illness, social influence and trust of the healthcare system, and knowledge and information about the vaccine (9–13). As a result, the availability of COVID-19 vaccines alone does not guarantee that people will readily receive vaccination.

Most countries across the world started the rollout of COVID-19 vaccination toward the last quarter of 2020. Consequently, it became important to examine people's willingness to get the COVID-19 vaccines. However, knowledge about people's willingness to get the COVID-19 vaccine was very limited even in developed countries. Perceptions and attitudes concerning the benefits and risks of vaccination are often premised on the claimed safety and efficacy of vaccines. Several rumors have been spread about COVID-19 vaccines since their development. These rumors have linked COVID-19 vaccines to various adverse effects such as infertility, reports of blood clots, several cases of death, immune thrombocytopenia, internal bleeding, low platelet counts, and cerebral venous thrombosis. These side effects have quite significantly affected vaccination campaigns in many countries (14–17).

As of February 26, 2022, there have been 742,541 confirmed cases of COVID-19 with 8,991 reported deaths in the KSA according to the World Health Organization (18). Several COVID-19 protocols and prevention measures such as social distancing, wearing masks, and using hand sanitizers have been put in place by the KSA public health authorities (19). However, vaccinating the population is one of the most effective ways to prevent the spread of COVID-19 and reduce its complications (20). Studies in several countries, including China, the United States, Italy, and Saudi Arabia, examined the willingness of people to accept the vaccine and the associated beliefs and barriers, showing that certain negative perceptions, beliefs, and attitudes are inhibiting some segments of the population from being vaccinated for COVID-19 (20–23).

Moreover, there is paucity of evidence about the side effects of COVID-19 vaccination and people's perceptions about COVID-19 vaccination in Saudi Arabia. Understanding side effects of COVID-19 vaccination and people's perception about COVID-19 vaccines will help to come up with effective interventions. The main aim of this study was to assess the side effects and perceptions about COVID-19 vaccines among Saudi Arabia's adult population following vaccination. Providing empirical evidence on the perceptions about COVID-19 vaccines and their side effects will be valuable in predicting the trends about future vaccine uptake and consequently developing strategies to improve acceptability (and uptake following vaccine availability).

## Materials and Methods

### Study Design

Data used for this study were derived from an online cross-sectional survey conducted between July 13 and July 20, 2021. The survey was self-administered using an online survey tool (SurveyMonkey Inc., San Mateo, CA, USA). Social media platforms such as Twitter and WhatsApp were used to send respondents invitations to participate in the study. The study was entirely conducted and reported according to Strengthening the Reporting of Observational Studies in Epidemiology (STROBE) guidelines for cross-sectional studies (24).

### Participants

The study conducted among individuals aged 18 years and older who had received one or both doses of the COVID-19 vaccines. All study participants had to be living in the KSA at the time of the survey. Participants were recruited for the survey using a simplified snowball sampling technique in which participants were asked to send on the invitations to their contacts. The online platform was used to minimize physical contact with study participants in line with COVID-19 protocols. Moreover, the online approach gave us the opportunity to gather information from as many respondents as possible.

Based on the latest KSA census, the population of Saudi Arabia was estimated to be 35,013,414 (25). From this population, a sample size calculator (26) was used to calculate the required sample for the study. A representative target sample of 1,037 participants, using a ±4% margin of error, confidence level of 99%, and 50% response distribution, was derived. A total of 1,094 participants successfully completed the questionnaire across the 13 regions of the KSA. Participants who lived outside Saudi Arabia at the time of completing the survey and those with missing data on variables of interest for the current study were excluded. Hence, the final sample included for analysis was 1,058 participants.

### Instrument

The self-reported questionnaire used for this study was adopted from previous studies and frameworks used to assess side effects of vaccines following vaccination (17, 27, 28). The questionnaire was assessed and validated by a panel of experts in medicine and infectious diseases that carefully reviewed the items of the questionnaire and provided feedback, which was used to further improve the questionnaire. The Cronbach alpha coefficient for the questionnaire was 0.75, indicating good internal validity (29).

There were four main sections of the questionnaire: (i) sociodemographic questions; (ii) health behavior, type of vaccination received, and knowledge and sources of information about COVID-19 vaccines; (iii) perceptions and beliefs following vaccination; and (iv) reactions after vaccination. The survey questionnaire was written in English and then translated to Arabic. The Arabic version was used to administrate the survey.

### Measurement of Variables

#### Dependent Variables

There were two major outcomes used for analysis: side effects and perceptions of COVID-19 vaccines. For side effects, the question used was: “following vaccination have you noticed any symptoms?” The response categories were: No symptoms at all (0); Yes, mild symptoms (1); Yes, moderate symptoms (2); and Yes, severe symptoms (3). These same categories were used for the multinomial logistic regression analysis. For perceptions about COVID-19 vaccinations, the following questions were used: “Do you think COVID-19 vaccines are safe in the long term?” “Are you monitoring your vital signs more frequently after vaccination?” and “Do you advise others to get vaccinated for COVID-19?” All of these questions were based on binary responses (yes = 1 and no = 0), and this same coding was maintained for binary logistic regression analysis.

#### Independent Variables

Sociodemographic characteristics of individuals were used as independent variables. These variables included: gender, age, educational level, employment status, and nationality. Health-related variables used were smoker, suffering from a chronic illness, number of vaccine doses received, infected with COVID-19 before vaccination, and feeling anxious about the COVID-19 vaccine before receiving it. Other variables used for descriptive analysis included preferred vaccine brand, source of information on vaccines, type of COVID-19 vaccines received, and side effects experienced. A description of the independent variables and their measurements are presented in Table 1.

**Table 1:** Independent variable specifications.

**Variables**	**Measurement**
**Sociodemographic**	
Gender	Whether the respondent is male or female; 0 for female, 1 for male
Age	Age of respondent; 18–29 (reference category), 30–39, 40–49, 50–59, ≥60 years.
Marital status	The marital status of the respondent; 0 for unmarried (single, widowed, or divorced), 1 for married.
Educational level	Educational level of the respondents; high school or below (reference category), university degree, post-graduate degree.
Employment status	Government sector employee (reference category), private sector employee, student, retired, unemployed.
Nationality	Whether the respondent is Saudi or non-Saudi; 0 for non-Saudi, 1 for Saudi.
**Health-related variables**	
Smoker	Whether the respondent is smoker; 0 for no, 1 for yes.
Chronic illness	Whether the respondent suffering from a chronic illness (diabetes mellitus, obesity, hypertension, chronic respiratory diseases, cardiovascular diseases, joint inflammation, autoimmune diseases, thyroid disorders, cancers, osteoporosis, and other chronic diseases), categorized as a binary variable; 0 for no, 1 for yes.
Number of vaccine doses received	0 for one dose, 1 for two doses.
Infected with COVID-19 before vaccination	Whether the respondent infected with COVID-19 before vaccination; 0 for no, 1 for yes.
Anxiety	Whether the respondent feeling anxious about the COVID-19 vaccine before receiving it; 0 for no, 1 for yes.
**Other variables**	
Preferred vaccine brand	No preference, AstraZeneca/Oxford, Pfizer/BioNTech, others including Moderna and Johnson and Johnson.
Source of information on vaccines	Government-owned media platforms, scientific and medical platforms, social media platforms, friends and relatives, no information.
Type of COVID-19 vaccines received	AstraZeneca/Oxford or Pfizer/BioNTech.
Side effects experienced	Whether the respondent noticed any symptoms after vaccination. These including: tiredness/fatigue, swelling, fever, headache, muscle pains, joint pains, sleepiness, dizziness, decreased sleep, nausea, chills, heart beats, cold, dry throat, haziness, dyspnea, body sweats, abdominal pain, irritation, chest pains, diarrhea, runny nose, bruises, blood pressure, vomiting, swollen feet, bleeding gums, and nose bleeding.

### Data Analysis

Data analysis for this study were conducted through descriptive statistics such as the frequency distribution using Statistical Package for the Social Sciences (SPSS) software, version 27.0. Descriptive statistics was used to assess the prevalence of side effects and perceptions about COVID-19 vaccines following vaccination. This was followed by a Pearson chi-square test (χ2) and multicollinearity test using the variance inflation factor. The multicollinearity test was performed to check for possible collinearity between the explanatory variables used in this study. Bivariate and multivariable logistic regression analyses were performed, followed by testing model fitness (Hosmer-Lemeshow, *p* = 0.3530). The multivariable logistic regression (for both binary and multinomial) results are presented as the adjusted odd ratio (aOR) and 95% confidence interval (CI). Given the sampling used for the survey and the complex nature of the data, we used the “complex samples” module in SPSS for these analyses.

## Results

### Sample Description

Table 2 shows the classification of participants involved in the study based on their demographic and health data. A slightly higher proportion of participants were females (51.0%), aged 30–39 years (34.3%), married (75.2%), university degree holders (43.5%), of Saudi nationality (93.2%), and government employees (51.1%). The percentage of individuals who reported being smokers was 24.6%, and 33.4% reported suffering from chronic conditions. Approximately half of the participants (49.2%) had taken two doses, 52.5% of participants reported that they were anxious about COVID-19 vaccines, and 14.4% indicated that they were infected with COVID-19 before vaccination.

**Table 2:** Classification of participants involved in the study based on their demographic and health data.

**Variable**	***N***	**%**
**Gender**		
Female	540	51.0
Male	518	49.0
**Age (years)**		
18–29	143	13.5
30–39	363	34.3
40–49	294	27.8
50–59	154	14.6
≥60	104	9.8
**Marital status**		
Unmarried	262	24.8
Married	796	75.2
**Educational level**		
High school or below	229	21.6
University degree	460	43.5
Postgraduate degree	369	34.9
**Employment status**		
Government sector employee	541	51.1
Private sector employee	161	15.2
Student	70	6.6
Retired	110	10.4
Unemployed	176	16.7
**Nationality**		
Non-Saudi	72	6.8
Saudi	986	93.2
**Smoker**		
No	798	75.4
Yes	260	24.6
**Chronic illness**		
No	705	66.6
Yes	353	33.4
**Number of doses received**		
One	538	50.9
Two	520	49.1
**Infected with COVID-19 before vaccination**		
No	906	85.6
Yes	152	14.4
**Anxiety**		
No	503	47.5
Yes	555	52.5
Total	1,058	100

### Pre-vaccination

#### Preferred Vaccine

**Figure 1:**
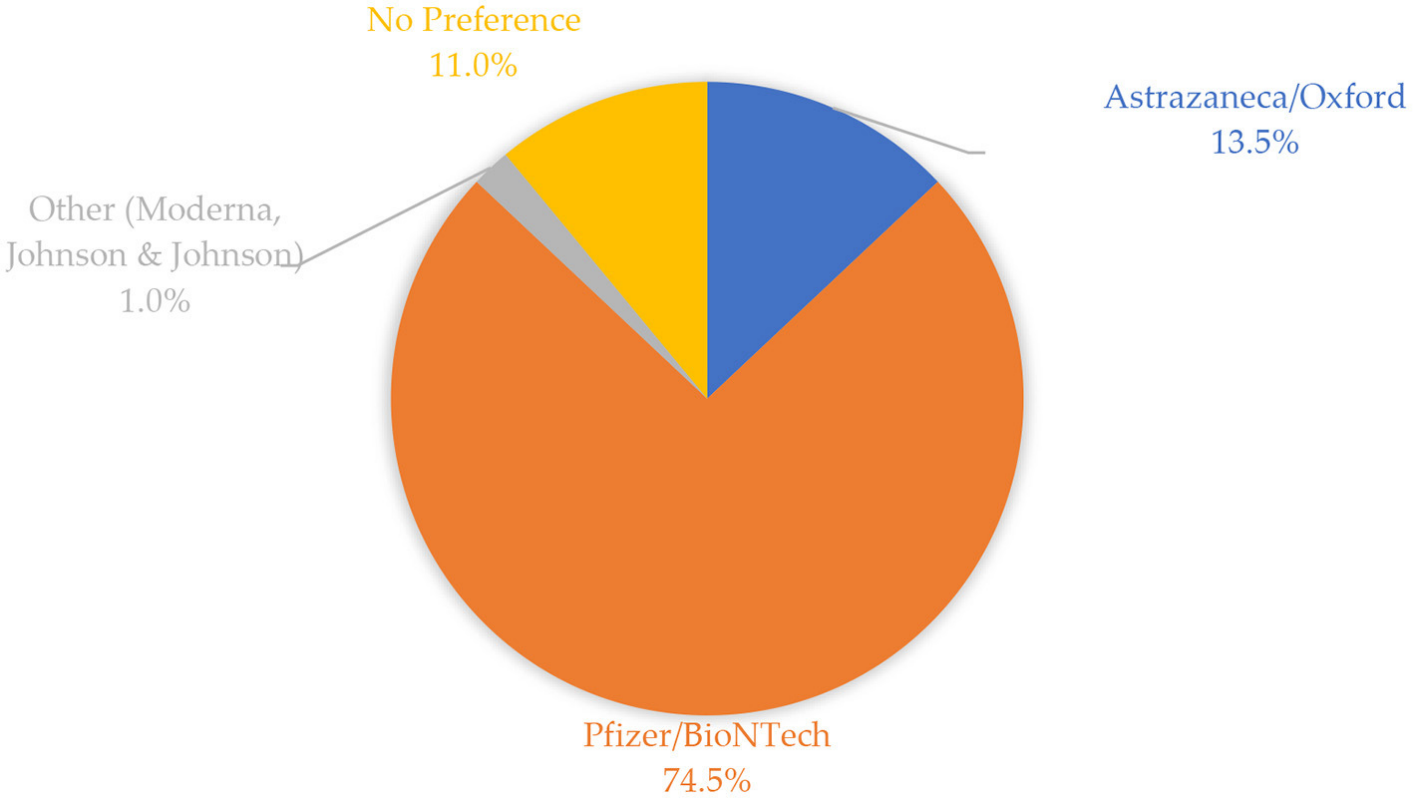
Frequencies of vaccines preferred by study participants.

Figure 1 shows the frequencies of vaccines preferred by study participants. A high proportion of study participants preferred receiving the Pfizer-BioNTech vaccine (74.5%), followed by AstraZeneca/Oxford (13.5%), and other vaccines such as Johnson & Johnson and Moderna (1%). The remaining proportion constituted participants who did not have any vaccine preference (11%).

#### Source of Information About COVID-19 Vaccination

**Figure 2:**
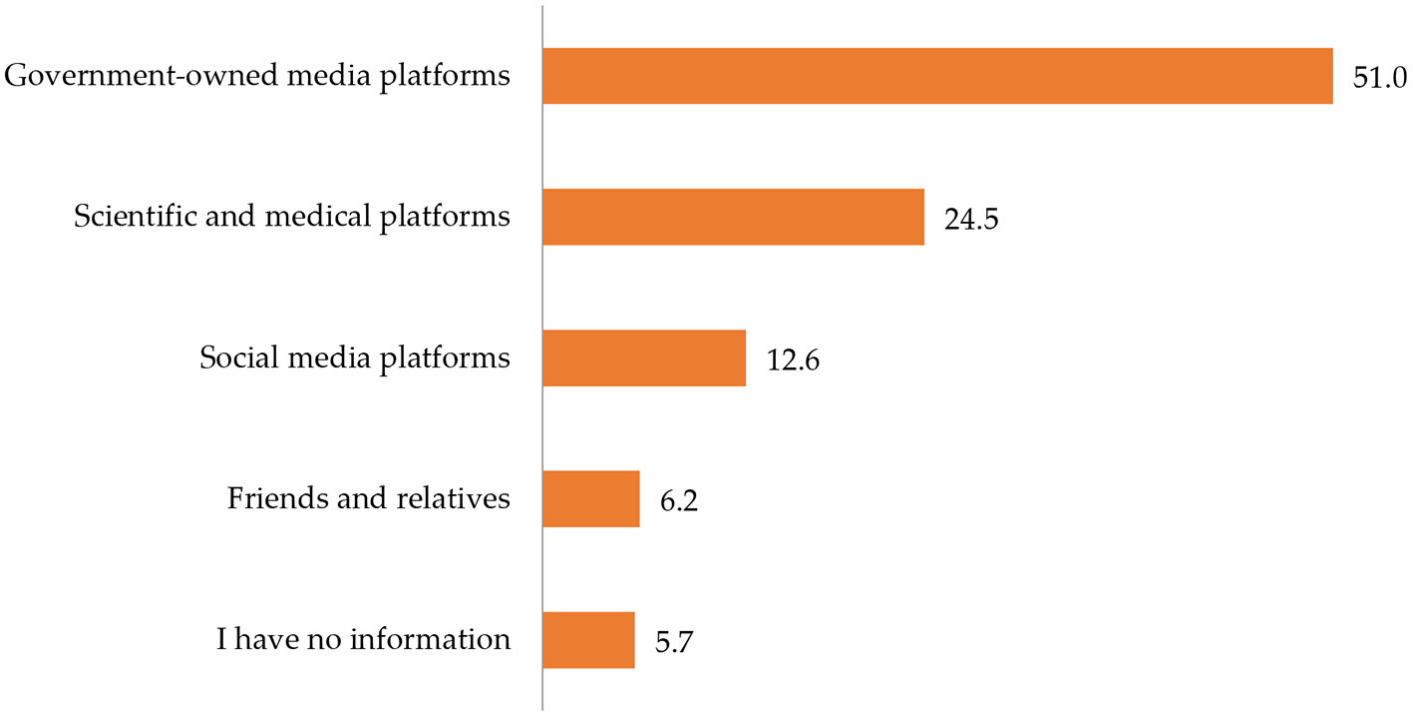
Source of information about COVID-19 vaccination.

Figure 2 indicates that slightly more than half (51.0%) of the participants obtained information about COVID-19 vaccines from government-owned media platforms, while 43.3% of the participants obtained information from other various sources such as social media platforms, friends and relatives, scientific and medical platforms.

### Type and Doses of COVID-19 Vaccine Taken by Participants

Table 3 shows the classification of participants based on the type of COVID-19 vaccine they received. Slightly over half (50.9%) of the respondents reported that they had received a single dose. From this proportion, over two thirds (66.9%) had received AstraZeneca/Oxford while over two-fifths (45.8%) had received Pfizer/BioNTech. Among those who had received two doses, a higher proportion had received Pfizer-BioNTech (54.2%) than AstraZeneca/Oxford (33.1%).

**Table 3:** Classification of participants based on types of COVID-19 vaccine received.

**Vaccine**	**One dose** ***n* (%)**	**Two doses** ***n* (%)**	**Total** **n (%)**
AstraZeneca/Oxford	170 (66.9)	84 (33.1)	254 (24.0)
Pfizer-BioNTech	368 (45.8)	436 (54.2)	804 (76.0)
Total	538 (50.9)	520 (49.1)	1,058 (100)

### Post-vaccination

#### Side Effects of COVID-19 Vaccination

**Figure 3:**
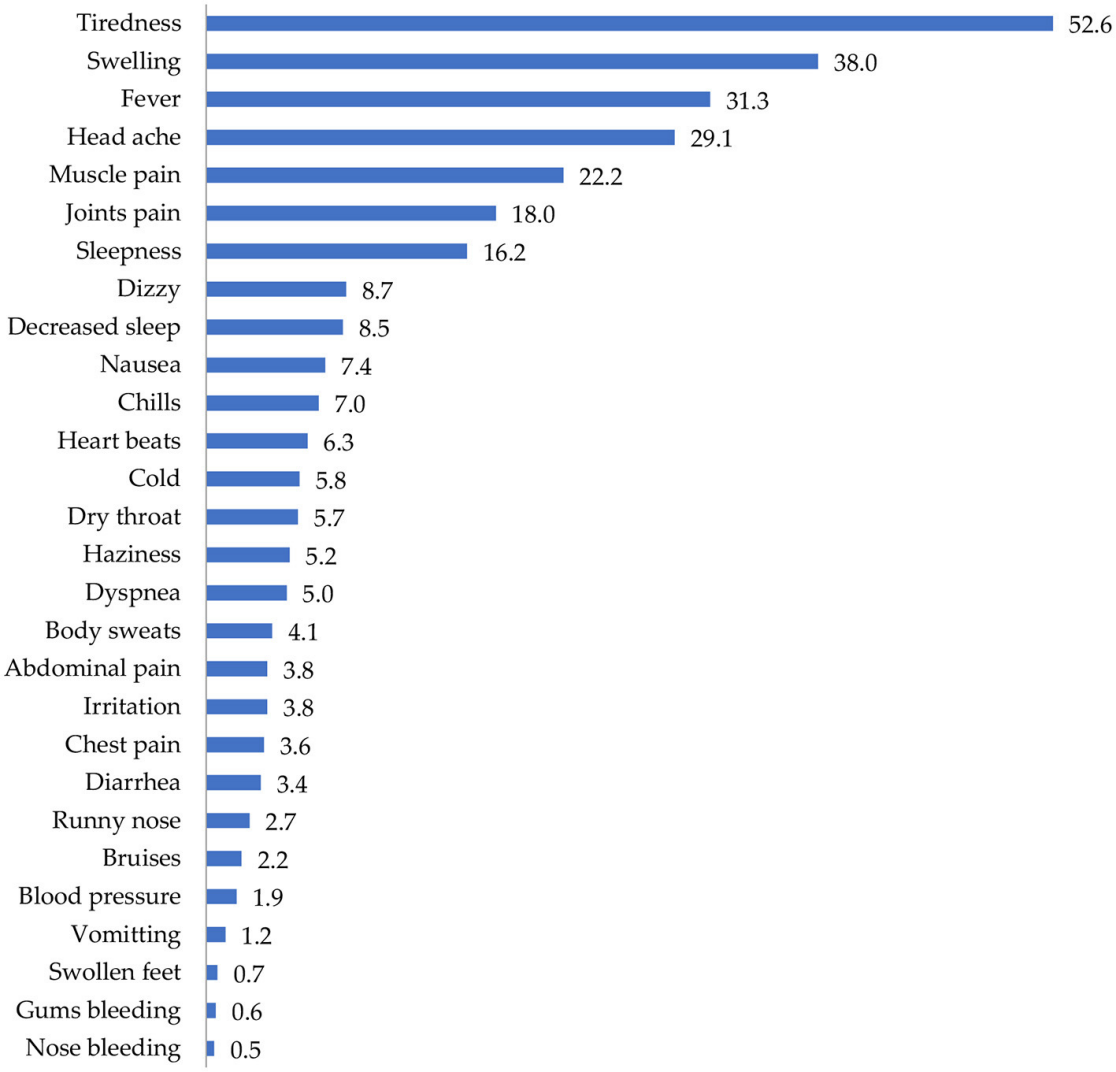
Side effects of COVID-19 vaccination in the sampled population.

Figure 3 shows the distribution of side effects from COVID-19 vaccines in the sampled population. More than half of the participants (52.6%) reported that they experienced tiredness/fatigue after vaccination with COVID-19 vaccines. Other side effects experienced included swelling (38%), fever (31.3%), headache (29.1%), and muscle pain (22.2%).

#### Severity of Side Effects After Vaccination

**Figure 4:**
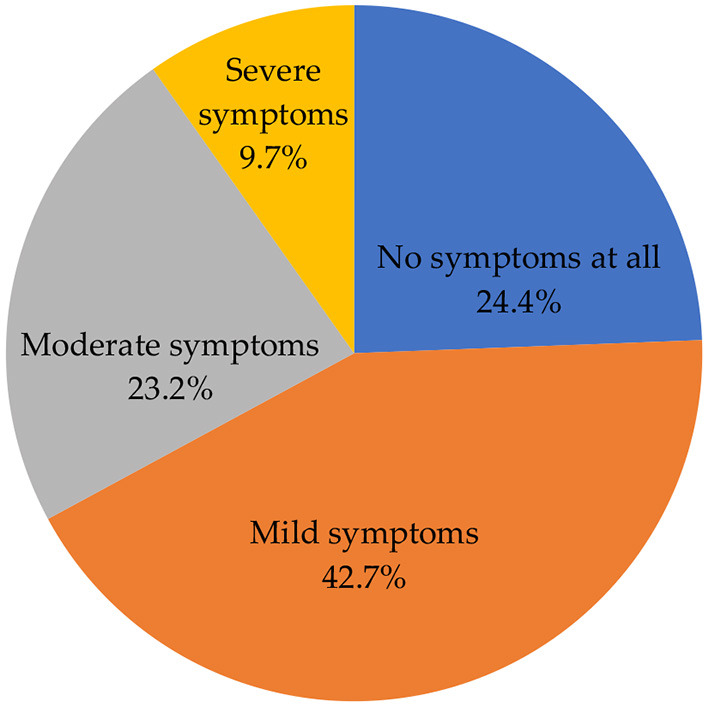
Severity of side effects after vaccination.

Figure 4 shows the severity of side effects after vaccination. A high proportion of participants indicated that they experienced mild symptoms (42.7%). The remaining proportion constituted those who experienced no symptoms at all (24.4%), moderate (23.2%), and severe (9.7%) symptoms.

Table 4 shows the results of the severity of side effects by the socio-demographic and health characteristics of participants, which were compared using the χ2 statistic. The severity of side effects is associated with gender (χ2 = 32.75, *p* <0.01). A slightly higher percentage among females than males reported mild (44.3 vs. 41.1%), moderate (25.7 vs. 20.5%), and severe (12.4 vs. 6.9%) symptoms. The severity of side effects was also associated with smoking status (χ2 = 17.63, *p* <0.01); a higher proportion of smokers experienced moderate symptoms compared to non-smokers (43.1 vs. 42.6%), while higher proportions of non-smokers experienced mild (25.2 vs. 16.9%) and severe (10.5 vs. 7.3%) symptoms than smokers.

**Table 4:** Participants' experience and severity of side effects by sociodemographic and health characteristics.

**Variable**	**Severity of side effects**					
	**No side effects *n* (%)**	**Mild *n* (%)**	**Moderate *n* (%)**	**Severe** ***n* (%)**	**χ^2^**	***P*-value**
**Gender**						
Female	95 (17.6)	239 (44.3)	139 (25.7)	67 (12.4)	32.75	<0.01
Male	163 (31.5)	213 (41.1)	106 (20.5)	36 (6.9)		
**Age (years)**						
18–29	30 (21.0)	69 (48.3)	29 (20.3)	15 (10.5)	17.98	0.12
30–39	88 (24.2)	150 (41.3)	85 (23.4)	40 (11.0)		
40–49	64 (21.8)	128 (43.5)	73 (24.8)	29 (9.9)		
50–59	36 (23.4)	64 (41.6)	41 (26.6)	13 (8.4)		
≥60	40 (38.4)	41 (39.4)	17 (16.3)	6 (5.8)		
**Marital status**						
Unmarried	60 (22.9)	120 (45.8)	55 (21.0)	27 (10.3)	1.91	0.59
Married	198 (24.9)	332 (41.7)	190 (23.9)	76 (9.5)		
**Educational level**						
High school or below	63 (27.5)	95 (41.5)	49 (21.4)	22 (9.6)	3.96	0.68
University degree	105 (22.8)	192 (41.7)	113 (24.6)	50 (10.9)		
Postgraduate degree	90 (24.4)	165 (44.7)	83 (22.5)	31 (8.4)		
**Employment status**						
Government sector employee	131 (24.2)	229 (42.3)	129 (23.8)	52 (9.6)	12.99	0.37
Private sector employee	37 (23.0)	74 (46.0)	35 (21.7)	15 (9.3)		
Student	13 (18.6)	31 (44.3)	18 (25.7)	8 (11.4)		
Retired	37 (33.6)	49 (44.5)	18 (16.4)	6 (5.5)		
Unemployed	40 (22.7)	69 (39.2)	45 (25.6)	22 (12.5)		
**Nationality**						
Non-Saudi	8 (11.1)	33 (45.8)	20 (27.8)	11 (15.3)	8.91	0.03
Saudi	250 (25.4)	419 (42.5)	225 (22.8)	92 (9.3)		
**Smoker**						
No	173 (21.7)	340 (42.6)	201 (25.2)	84 (10.5)	17.63	<0.01
Yes	85 (32.7)	112 (43.1)	44 (16.9)	19 (7.3)		
**Chronic illness**						
No	177 (25.1)	304 (43.1)	161 (22.8)	63 (8.9)	2.01	0.571
Yes	81 (22.9)	148 (41.9)	84 (23.8)	40 (11.3)		
**Number of doses received**						
One	131 (24.3)	217 (40.3)	131 (24.3)	59 (11.0)	3.84	0.28
Two	127 (24.4)	235 (45.2)	114 (21.9)	44 (8.5)		
**Infected with COVID-19 before vaccination**						
No	220 (24.3)	396 (43.7)	204 (22.5)	86 (9.5)	2.96	0.40
Yes	38 (25.0)	56 (36.8)	41 (27.0)	17 (11.2)		
**Anxiety**						
No	141 (28.0)	214 (42.5)	107 (21.3)	41 (8.2)	9.18	0.03
Yes	117 (21.1)	238 (42.9)	138 (24.9)	62 (11.2)		
Total	258 (24.4)	452 (42.7)	245 (23.2)	103 (9.7)		

Moreover, moderate (42.9 vs. 42.5%), mild (24.9 vs. 21.3%), and severe (11.2 vs. 8.2%) side effects were also observed to be higher among individuals who reported that they felt anxious about the COVID-19 vaccine compared to those who were not anxious (χ2 = 9.18, *p* = 0.03). However, there was no statistically significant association observed between the severity of side effects and age, marital status, educational level, employment status, number of doses, whether one had a chronic condition, and whether they were infected with COVID-19 before vaccination.

#### Logistic Regression Analyses for the Severity of Side Effects

Table 5 shows the results of logistic regression analyses for the severity of side effects. The odds of experiencing severe side effects were significantly higher among males (aOR = 2.76, 95% CI = 1.71–4.45, *p* <0.01) than females. For age, the odds of experiencing mild side effects were significantly higher among people aged 30–39 years (aOR = 3.52, 95% CI = 1.61–7.68), 40–49 years (aOR = 2.44, 95% CI = 1.31–4.57, *p*<0.05), 50–59 years (aOR = 2.63, 95% CI = 1.41–4.92, *p* <0.05), and 60 years and above (aOR = 1.94, 95% CI = 1.08–3.48, *p* <0.1) compared to individuals aged 18–29 years. Saudi nationals had significantly higher odds of reporting mild (aOR = 2.44, 95% CI = 1.23–4.86, *p* <0.05), moderate (aOR = 2.65, 95% CI = 1.27–2.71, *p* <0.05), and severe (aOR = 3.64, 95% CI = 1.58–8.38, *p* <0.05) side effects compared to non-Saudis.

**Table 5:** Logistic regression analysis results for the severity of side effects.

	**Severity of side effects** ^**†**^		
**Variable**	**Mild** **aOR (95% CI)**	**Moderate** **aOR (95% CI)**	**Severe** **aOR (95% CI)**
**Gender**			
Female (ref)			
Male	1.93 (1.40–2.66)***	1.97 (1.37–2.84)***	2.76 (1.71–4.45)***
**Age (years)**			
18–29 (ref)			
30–39	3.52 (1.61–7.68)***	1.90 (0.74–4.84)	2.99 (0.86–10.3)
40–49	2.44 (1.31–4.57)**	2.19 (1.05–4.59)*	3.10 (1.10–8.72)*
50–59	2.63 (1.41–4.92)**	2.34 (1.12–4.88)*	2.61 (0.93–7.36)
≥60	1.94 (1.08–3.48)*	2.19 (1.09–4.38)*	1.83 (0.67–4.97)
**Marital status**			
Unmarried (ref)			
Married	0.90 (0.62–1.32)	0.76 (0.49–1.18)	0.80 (0.46–1.39)
**Educational level**			
High school or below (ref)			
University degree	0.81 (0.55–1.20)	0.83 (0.53–1.30)	1.03 (0.56–1.87)
Postgraduate degree	0.95 (0.69–1.30)	1.20 (0.83–1.73)	1.42 (0.87–2.31)
**Employment status**			
Government sector employee (ref)			
Private sector employee	1.58 (0.99–2.51)	1.44 (0.86–2.40)	1.64 (0.87–3.09)
Student	1.70 (1.01–2.85)*	1.35 (0.75–2.43)	1.39 (0.67–2.89)
Retired	1.66 (0.78–3.55)	2.64 (1.11–6.27)*	2.21 (0.76–6.37)
Unemployed	2.03 (1.23–4.86)*	1.04 (0.48–2.25)	1.10 (0.37–3.22)
**Nationality**			
Non-Saudi (ref)			
Saudi	2.44 (1.23–4.86)**	2.65 (1.27–2.71)**	3.64 (1.58–8.38)**
**Smoker**			
No (ref)			
Yes	1.34 (0.98–1.82)	1.86 (1.27–2.71)***	1.64 (0.98–2.73)
**Chronic illness**			
No (ref)			
Yes	0.76 (0.56–1.05)	0.68 (0.47–0.98)*	0.48 (0.30–0.75)**
**Number of doses received**			
One (ref)			
Two	0.72 (0.53–0.98)*	0.83 (0.58–1.18)	0.87 (0.55–1.36)
**Infected with COVID-19 before vaccination**			
No (ref)			
Yes	1.08 (0.72–1.61)	0.83 (0.54–1.29)	0.87 (0.49–1.53)
**Anxiety**			
No (ref)			
Yes	0.83 (0.63–1.09)	0.76 (0.55–1.05)	0.71 (0.47–1.08)

*** *p <0.01,*

** *p <0.05,*

* *p <0.1; aOR, adjusted odd ratios, CI, confidence interval*.

† *No side effects is the reference category in the multinomial logistic regression model*.

Considering employment status, the odds of reporting mild side effects were significantly higher among students (aOR = 1.70, 95% CI = 1.01–2.86, *p* <0.1) and unemployed individuals (aOR = 2.03, 95% CI = 1.23–4.86, *p* <0.1) compared to government employees, whereas the odds of reporting moderate side effects were significantly higher among retired individuals (aOR = 2.64, 95% CI = 1.11–6.27, *p* <0.1) compared to government employees. Conversely, individuals who were smokers (aOR = 1.86, 95% CI = 1.27–2.71, *p* <0.01) had higher odds of reporting moderate side effects compared to those who did not smoke. The odds of reporting moderate (aOR = 0.68, 95% CI = 0.47–0.98, *p* <0.1) and severe (aOR = 0.48, 95% CI = 0.30–0.75, *p* <0.05) side effects were significantly lower among individuals who indicated that they suffer from chronic illnesses compared to their counterparts. Individuals who had received two doses (aOR = 0.72, 95% CI = 0.53–0.98, *p* <0.1) also had lower odds of reporting mild side effects compared to those who had received one dose.

### Perceptions About COVID-19 Vaccines

#### Participants' Perceptions About COVID-19 Vaccines by Sociodemographic and Health Characteristics: Bivariate Association Analysis

Table 6 shows the bivariate association between participants' perceptions about COVID-19 vaccines by demographic and health characteristics. A significantly high proportion among males (66.8%), high school or below participants (62.0%), retired individuals (67.3%), smokers (62.7%), those who had received two doses (64.2%), and those who reported that they did not feel anxious about the COVID-19 vaccine before receiving it believed that COVID 19 vaccines are safe in the long-term. However, only a significantly higher percentage among married (51.5%) respondents compared to unmarried (45.4%) respondents reported that monitoring signs became more frequent after vaccination.

**Table 6:** Participants' perceptions about COVID-19 vaccines after vaccination by demographic and health characteristics.

**Variable**	**Believing that COVID-19 vaccines are safe in the long-term** ***n* (%)**	**Monitoring signs became more frequent after vaccination** ***n* (%)**	**Advise others to get vaccinated for COVID-19** ***n* (%)**
**Gender**			
Female	254 (47.0)	257 (47.6)	431 (79.8)
Male	346 (66.8)***	272 (52.5)	459 (88.6)***
**Age (years)**			
18–29	76 (53.1)	70 (49.0)	117 (81.8)
30–39	200 (55.1)	169 (46.6)	298 (82.1)
40–49	165 (56.1)	153 (52.0)	248 (84.4)
50–59	89 (57.8)	78 (50.6)	134 (87.0)
≥60	70 (67.3)	59 (56.7)	93 (89.4)
**Marital status**			
Unmarried	143 (54.6)	119 (45.4)*	213 (81.3)
Married	457 (57.4)	410 (51.5)	677 (85.1)
**Educational level**			
High school or below	142 (62.0)*	121 (52.8)	197 (86.0)
University degree	244 (53.0)	236 (51.3)	379 (82.4)
Postgraduate degree	214 (58.0)	172 (46.6)	314 (85.1)
**Employment status**			
Government sector employee	313 (57.9)***	264 (48.8)	458 (84.7)***
Private sector employee	88 (54.7)	78 (48.4)	136 (84.5)
Student	46 (65.7)	36 (51.4)	60 (85.7)
Retired	74 (67.3)	65 (59.1)	103 (93.6)
Unemployed	79 (44.9)	86 (48.9)	133 (75.6)
**Nationality**			
Non-Saudi	37 (51.4)	36 (50.0)	64 (88.9)
Saudi	563 (57.1)	493 (50.0)	826 (83.8)
**Smoker**			
No	437 (54.8)	401 (50.3)	658 (82.5)
Yes	163 (62.7)**	128 (49.2)	232 (89.2)
**Chronic illness**			
No	398 (56.5)	343 (48.7)	591 (83.8)
Yes	202 (57.2)	186 (52.7)	299 (84.7)
**Number of doses received**			
One	266 (49.4)***	261 (48.5)	412 (76.6)***
Two	334 (64.2)	268 (51.5)	478 (91.9)
**Infected with COVID-19 before vaccination**			
No	510 (56.3)	461 (50.9)	770 (85.0)
Yes	90 (59.2)	68 (44.7)	120 (78.9)*
**Anxiety**			
No	380 (75.5)	257 (51.1)	476 (94.6)
Yes	220 (39.6)***	272 (49.0)	414 (74.6)***

*** *p <0.01,*

** *p <0.05,*

* *p <0.1*.

A significantly higher proportion of males (88.6%) than females (79.8%) reported that they would advise others to get vaccinated for COVID-19. Similarly, a significantly higher proportion among students (85%), individuals who had taken two doses (91.9%), those not infected with COVID-19 before vaccination (85%), and those who indicated that they did not feel anxious about the COVID-19 vaccine before receiving it (94.6%) reported that they would advise others to get vaccinated for COVID-19 compared to their counterparts.

#### Logistic Regression of Participant's Perceptions About COVID-19 Vaccines After Vaccination

Table 7 shows the results of the logistic regression analyses for participants' perceptions about COVID-19 vaccines after vaccination. After adjusting for covariates, the odds of believing that COVID-19 vaccines are safe in the long-term were significantly higher among males (aOR = 1.76, 95% CI = 1.116–2.65, *p* <0.01) than females. Similarly, the odds of believing that COVID-19 vaccines are safe in the long-term were significantly higher among individuals who had received two doses (aOR = 1.62, 95% CI = 1.09–2.40, *p* <0.05) compared to those who had received only one dose. Moreover, individuals who were feeling anxious about the COVID-19 vaccine before receiving it (aOR = 0.24, 95% CI = 0.17–0.35, *p* <0.01) had lower odds of believing that COVID-19 vaccines are safe in the long-term.

**Table 7:** Logistic regression analyses for participants' perceptions about COVID-19 vaccines after vaccination broken down by sociodemographic and health characteristics.

**Variable**	**Believing that COVID-19 vaccines are safe in the long-term** **aOR (95% CI)**	**Monitoring signs became more frequent after vaccination** **aOR (95% CI)**	**Advise others to get vaccinated for COVID-19** **aOR (95% CI)**
**Gender**			
Female (ref)			
Male	1.76 (1.16–2.65)***	1.19 (0.81–1.74)	1.22 (0.70–2.12)
**Age (years)**			
18–29 (ref)			
30–39	1.32 (0.62–2.79)	0.92 (0.46–1.81)	0.87 (0.34–2.22)
40–49	1.40 (0.50–2.98)	1.09 (0.53–2.23)	0.92 (0.34–2.50)
50–59	1.22 (0.50–2.98)	0.90 (0.38–2.03)	0.79 (0.25–2.50)
≥60	1.20 (0.40–3.53)	1.01 (0.38–2.69)	0.44 (0.10–1.88)
**Marital status**			
Unmarried (ref)			
Married	0.96 (0.59–1.57)	1.28(0.81-2.01)	1.24(0.67-2.30)
**Educational level**			
High school or below (ref)			
University degree	0.74 (0.46–1.20)	0.96 (0.61–1.48)	0.75 (0.39–1.45)
Postgraduate degree	0.89 (0.52–1.51)	0.77 (0.47–1.24)	0.91 (0.44–1.89)
**Employment status**			
Government sector employee (ref)			
Private sector employee	0.98 (0.56–1.70)	0.9 (0.59–1.61)	0.99 (0.47–2.07)
Student	2.37 (0.87–6.42)	1.23 (0.51–2.98)	1.45 (0.39–5.30)
Retired	1.00 (0.44–2.29)	1.30 (0.58–1.70)	2.07 (0.52–8.18)
Unemployed	1.08 (0.60–1.92)	1.00 (0.58–1.70)	0.85 (0.42–1.73)
**Nationality**			
Non-Saudi (ref)			
Saudi	1.16 (0.56–2.41)	0.95 (0.49–1.83)	0.49 (0.17–1.44)
**Smoker**			
No (ref)			
Yes	1.03 (0.67–1.61)	0.60 (0.60–1.34)	1.37 (0.72–2.61)
**Chronic illness**			
No (ref)			
Yes	1.01 (0.67–1.52)	1.12 (0.77–1.63)	1.02 (0.59–1.74)
**Number of doses received**			
One (ref)			
Two	1.62 (1.09–2.40)**	1.01 (0.70–1.46)	2.81 (1.60–4.93)***
**Infected with COVID-19 before vaccination**			
No (ref)			
Yes	1.21 (0.72–2.05)	0.77 (0.48–1.25)	0.79 (0.42–1.49)
**Anxiety**			
No (ref)			
Yes	0.24 (0.17–0.35)***	0.97 (0.69–1.36)	0.19 (0.10–0.35)***

*** *p <0.01,*

** *p <0.05,*

* *p <0.1; aOR, adjusted odds ratio; CI, confidence interval*.

Conversely, there was no statistically significant association between the perception that monitoring signs became more frequent after vaccination and participants' sociodemographic and health characteristics. The odds of advising others to get vaccinated for COVID-19 were significantly higher among respondents who had received two doses (aOR = 2.81, 95% CI = 1.60–4.93, *p* <0.01) compared to their counterparts. By contrast, individuals who indicated that they were feeling anxious about the COVID-19 vaccine before receiving had significantly lower odds of advising others to get vaccinated for COVID-19 compared to those who did not feel anxious about the COVID-19 vaccine before receiving it.

## Discussion

The aim of this study was to assess the side effects and perceptions about COVID-19 vaccines among Saudi Arabia's adult population following vaccination. The findings indicate that a high proportion of participants preferred the Pfizer-BioNTech vaccine compared to AstraZeneca/Oxford and other vaccine types offered. As a result, most participants in this study were vaccinated with Pfizer-BioNTech (76.0%) and AstraZeneca/Oxford (24.0%). The preference for Pfizer-BioNTech and AstraZeneca/Oxford vaccines among study participants likely had more to do with vaccine availability, at the time of the study, than personal choice. Approximately half (50.9%) of the participants had taken the first dose at the time of completing the survey, with the remaining proportion constituting those who had received two doses (49.1%).

About than half of the participants indicated that they obtained information about COVID-19 vaccines from government-owned media platforms, with the remaining half obtaining relevant information from various sources such as social media platforms, friends and relatives, and scientific and medical platforms. To avoid misinformation about the pandemic and to ensure that people access accurate information, the Ministry of Health (MOH) of the KSA acted as a main and official source responsible for communicating COVID-19 information to the public (30). The dissemination of information has been implemented through engaging the community, using traditional channels such as television and text messages, as well as technology and digital health platforms. Moreover, the MOH developed high-quality media materials to be distributed and government-coordinated press conferences providing updates have been held on a daily basis during this pandemic. Furthermore, government leaders such as ministers and other prominent public figures have shared videos recommending that the public follow precautionary measures (30).

In this study, slightly more than two-fifths (42.7%) of the participants indicated that they experienced mild symptoms, with more than one-fifth (23.2%) and approximately one-tenth (9.7%) of the participants reporting that they experienced moderate and severe symptoms, respectively. Consistent with other studies in other countries, mild to moderate symptoms were the most common side effects reported following COVID-19 vaccination in our study (31, 32). Severe side effects were reported in almost one-tenth of the participants, which is similar to previous studies indicating that severe side effects are experienced by less than one-tenth of the vaccinated population (33, 34).

The most common types of side effects experienced by the study participants included tiredness/fatigue, swelling, fever, headache, muscle pains, joint pains, sleepiness, dizziness, decreased sleep, nausea, chills, heart beats, cold, dry throat, haziness, dyspnea, body sweats, abdominal pain, irritation, chest pains, diarrhea, runny nose, blood pressure, vomiting, swollen feet, bleeding gums, and nose bleeding. Similar findings have been observed in countries where Pfizer-BioNTech and AstraZeneca/Oxford vaccines have been used. For instance, injection fatigue and headache were the most common side effects reported in a several similar studies (35–37).

After adjusting for covariates, the odds of experiencing severe side effects were found to be significantly higher among males than among females. This is in contrast with the findings of the majority of previous studies showing that women have higher odds of reporting COVID-19 vaccine side effects compared to men, especially headache and fatigue (38). It has been argued that women are more likely to report their symptoms than men (39–41). Our findings thus provide important insights about the gendered dimensions of vaccination in the KSA. Therefore, there is a need to further investigate why men experienced and reported COVID-19 vaccination side effects more often than women in Saudi Arabia.

We also found that smokers were more likely to report having experienced moderate side effects compared to non-smokers. Previous investigations have also shown that smokers are more likely to experience some side effects (42). Since smoking is a health hazard, this can provide a plausible explanation for this finding. In particular, smoking is a common risk factor for most respiratory infections and has been noted to increases the severity of respiratory diseases. As a result, smokers are more likely to develop side effects after COVID-19 vaccination compared to non-smokers due to the weakened immune system (43).

With respect to age, the odds of experiencing severe side effects were significantly higher among people aged 30 years and above compared to those aged 18–29 years. This finding is consistent with results from several studies about COVID-19 vaccine side effect (38). The plausible explanation is that vaccine reactogenicity has been linked to raising of inflammatory cytokines, which shows that the vaccine reactogenicity declines with age, although it is not considered a reliable sign of a desirable immune response (44).

The study also found that individuals who reported that they were suffering from chronic illnesses were less likely to report moderate and mild side effects compared to those who did not report any chronic illness. There was no statistically significant association between reporting suffering from chronic illnesses and experiencing severe side effects after vaccination. There is little information from studies about COVID-19 vaccines to explain this observation. Although chronic diseases generally weaken the immune system and are more likely to create complications from COVID-19, which may lead to long-term illness, hospitalization, and even death, recent clinical trials show that COVID-19 vaccines are safe and effective among people with underlying medical conditions (28).

With respect to perceptions about COVID-19 vaccines after vaccination, after adjusting for covariates, the odds of believing that COVID-19 vaccines are safe in the long-term were significantly higher among men than among women. Similarly, the odds of believing that COVID-19 vaccines are safe in the long-term were significantly higher among individuals who had received two doses compared to those who had received only one dose. Although there is a dearth of evidence to ascertain this observation, this finding is quite indicative, and suggests that the experience or perception of safety and reduced risk of exposure to COVID-19 in the future, along with confidence in the vaccine may have collectively influenced the view of men and people who had received two doses that COVID-19 vaccines are safe in the long-term. As would be expected, individuals who were feeling anxious about the COVID-19 vaccine before receiving it had lower odds of believing that COVID-19 vaccines are safe in the long-term.

Quite conversely, there was no statistically significant association between the perception that monitoring signs became more frequent after vaccination and participants' sociodemographic and health characteristics. The odds of advising others to get vaccinated for COVID-19 were significantly higher among respondents who had received two doses compared to their counterparts. This finding agrees with other previous studies showing that being fully vaccinated is vital, given that infections are often mild or asymptomatic after receiving two doses of the vaccine (45–47). Being fully vaccinated was noted in preventing infection with SARS-CoV-2 variants by at least 50% (48), and this is expected to motivate those who are fully vaccinated to recommend the vaccine to others.

There are some limitations of this study. This was an online cross-sectional study, which also used the snowball sampling technique for recruitment that might have impacted the generalizability and affected the representativeness of the sample. As a result, our findings may not be representative of the opinions of people who live in areas where there is limited internet connectivity. However, an online cross-sectional survey was the only viable study design to be employed at the time of the survey due to social distance requirements. Another main limitation of this study was the use of a non-standardized questionnaire to collect the data.

Nevertheless, our findings will have several implications. First, our study can provide vital insights on people's perceptions and attitudes after receiving COVID-19 vaccines. This will in turn help in the design of effective behavior change communication campaigns by the healthcare system to dispel negative vaccination perceptions. Second, although there have been several studies on COVID-19 vaccine hesitancy published to date, there is a paucity of studies on the side effects of the vaccines experienced by the Saudi population. Therefore, our results will enlighten healthcare professionals and policymakers to address the perceptions and concerns regarding vaccinations and their side effects. The findings of this study are not only important at this current point in the COVID-19 pandemic and vaccine rollout but can further be used as a reference for policy effort in facing possible future epidemics. In particular, the information derived from this study can be used to educate the public regarding the importance of vaccination, side effects notwithstanding.

## Conclusions

This study provides evidence about the side effects and perceptions of COVID-19 vaccines among adults in Saudi Arabia. Common side effects reported by participants were tiredness/fatigue, swelling, fever, headache, muscle pains, joint pains, dizziness, decreased sleep, nausea, chills, heart beats, cold, dry throat, haziness, dyspnea, body sweats, abdominal pain, irritation, chest pains, diarrhea, and runny nose. These symptoms were linked to the two vaccine types predominately used in Saudi Arabia: Pfizer-BioNTech and AstraZeneca/Oxford. The odds of reporting severe side effects after vaccination were significantly higher among men, people aged 40–49 years, and Saudi nationals compared to their respective counterparts. Regarding the perceptions about COVID-19 vaccines, the odds of believing that COVID-19 vaccines are safe in the long-term were significantly higher among men and among individuals who had received two doses, and the odds of advising others to get vaccinated for COVID-19 were also significantly higher among respondents who had received two doses compared to their counterparts.

## Data Availability Statement

The datasets generated and/or analyzed during the current study are not publicly available due to privacy and confidentiality agreements as well as other restrictions but are available from the corresponding author on reasonable request.

## Ethics Statement

All procedures performed in this study involving human participants complied with the institutional and/or national research committee ethical standards and the 1964 Helsinki Declaration and subsequent amendments or equivalent ethical standards. The study was designed and conducted according to the ethical principles established by King Abdulaziz University. Ethical approval was obtained from the Biomedical Ethics Research Committee, Faculty of Medicine, King Abdulaziz University (Ref-380-21). The patients/participants provided online informed consent to participate in this study.

## Author Contributions

MKA and MK: conceptualization, data curation, formal analysis, software, validation, and writing—original draft preparation. MKA: methodology, project administration, supervision, and funding acquisition. MKA, MK, NK, NA, AQ, and OA: writing—review and editing. All authors have read and agreed to the published version of the manuscript.

## Funding

This research was funded by the Institutional Fund Projects under grant number IFPRC-207-121-2020. The funders had no role in the study design, data collection and analysis, decision to publish, or preparation of the manuscript.

## Conflict of Interest

The authors declare that the research was conducted in the absence of any commercial or financial relationships that could be construed as a potential conflict of interest.

## Publisher's Note

All claims expressed in this article are solely those of the authors and do not necessarily represent those of their affiliated organizations, or those of the publisher, the editors and the reviewers. Any product that may be evaluated in this article, or claim that may be made by its manufacturer, is not guaranteed or endorsed by the publisher.
